# The Role of Advanced Technologies and Artificial Intelligence (AI) in Surgical Training: A Consensus Report

**DOI:** 10.7759/cureus.98371

**Published:** 2025-12-03

**Authors:** S Vincent Grasso, Gaya Spolverato, Giulia Capelli, Daunia Verdi, Niki Rashidian, Tomilola Olakunde, Karol Rawicz Pruszynski, Isabella Frigerio, Roland Croner, Stephane Romano, Elie Chouillard, Andrew A Gumbs

**Affiliations:** 1 Department of Medical Education and Scholarship, Rowan-Virtua School of Osteopathic Medicine, Stratford, USA; 2 Department of Electrical and Computer Engineering, University of New Mexico, Albuquerque, USA; 3 Department of Surgical Oncology, Medical University of Lublin, Lublin, POL; 4 Department of Surgical, Oncological, and Gastroenterological Sciences, University of Padova, Padova, ITA; 5 Department of Surgery, ASST Bergamo EST, Bergamo, ITA; 6 Department of Surgery, Mirano Hospital, Mirano, ITA; 7 Department of Hepatobiliary Surgery and Liver Transplantation, Ghent University Hospital, Ghent, BEL; 8 Internal Medicine, Center for Translation and Implementation Research, Nsukka, NGA; 9 Department of Surgery, HPB Unit, Pederzoli Hospital, Peschiera del Garda, ITA; 10 Collegium Medicum, University of Social Sciences, Lodz, POL; 11 Department of General, Visceral, Vascular, and Transplantation Surgery, University of Magdeburg, Magdeburg, DEU; 12 Department of Orthopedic Surgery and Traumatology, American Hospital of Paris, Neuilly, FRA; 13 Department of Surgery, American Hospital of Paris, Neuilly, FRA; 14 Minimally Invasive Digestive Surgery Service, Hôpital Antoine Béclère-Assistance Publique Hopitaux de Paris, Clamart, FRA

**Keywords:** artificial intelligence, consensus, healthcare technology, medical simulation, surgical education, surgical training, telemedicine, telesurgery

## Abstract

This white paper presents the consensus opinions of the Artificial Intelligence Surgery (AIS) Editorial Board and its Task Force on Surgical Training regarding the integration and role of advanced technologies and artificial intelligence (AI) in surgical education. Derived from a structured modified Delphi process involving comprehensive video conference discussions and a 25-item questionnaire, the paper explores five critical topics: 1) Telemedicine, Telementoring, Telepresence, and Remote Surgery (Telesurgery); 2) The Role of Simulation in Surgical Education; 3) Technology's Role in Training Surgeons in Low- and Middle-Income Countries (LMICs); 4) Technology's Role in Training Disabled Surgeons; and 5) Ethical Aspects of AI-Based Surgical Training. The consensus process, conducted with 23 international experts, achieved ≥80% agreement on 13 of 25 statements. These consensus principles affirm the essential role of advanced technologies - including AI, simulation, and telementoring - in addressing the global surgical burden and guiding future developments for a more inclusive surgical workforce.

## Introduction

The World Health Organization (WHO) and The Lancet have repeatedly underscored a significant, unaddressed global surgical burden, with an estimated five billion people lacking access to safe, affordable, and timely surgical and anesthesia care [1,2]. This disparity is most pronounced in low- and middle-income countries (LMICs), where a critical shortage of trained surgeons persists [1].

Challenges of traditional training models

In these settings, traditional surgical training models are increasingly viewed as insufficient to meet this global need, primarily due to their resource-intensive nature, reliance on limited local patient volumes, chronic scarcity of expert mentorship, and lack of standardized curricula. The coronavirus disease 2019 (COVID-19) pandemic further exacerbated these challenges, accelerating the demand for innovative, technology-driven solutions to maintain and improve surgical education.

Adaptive and inclusive training

The challenges of traditional surgical training are not limited to resource constraints. The personal account of one of the authors, a surgeon who completed their training while recovering from a broken neck, serves as a case-in-point illustration of the personal resilience required and the potential for a career-ending injury to disrupt a surgeon's journey. This personal narrative underscores the need for training methods that are not only effective but also adaptive and inclusive, ensuring that qualified individuals can contribute to the surgical workforce regardless of physical limitations. Advanced technologies may provide the necessary tools to overcome such barriers, offering a pathway for surgeons with disabilities to continue their invaluable contributions to the field.

Technology as a solution

Advanced technologies such as virtual reality (VR), augmented reality (AR), telementoring, and artificial intelligence (AI) offer a powerful pathway to transform surgical training [3,4,5]. These technologies can create safe, repeatable, and objective learning environments, allowing trainees to acquire and refine skills without risk to patients [6]. While these innovations are becoming commonplace in high-income countries (HICs), a unified global strategy for their adoption, one that specifically addresses the unique challenges and opportunities in LMICs, remains underdeveloped. This lack of a global strategy is primarily due to profound barriers in LMICs, including unstable infrastructure, high equipment cost, limited internet connectivity, and the absence of clear regulatory frameworks.

Primary objective and critical topics

The primary objective of this study was not to test a traditional hypothesis, but rather to establish a global consensus among international surgical and policy experts on the role, utility, and implementation of advanced technologies in surgical training, encompassing the following five critical topics: 1) Telemedicine, Telementoring, Telepresence, and Remote Surgery (Telesurgery). 2) The Role of Simulation in Surgical Education. 3) Technology's Role in Training Surgeons in Low- and Middle-Income Countries (LMICs). 4) Technology's Role in Training Disabled Surgeons. 5) Ethical Aspects of AI-Based Surgical Training.

Through a structured modified Delphi consensus process involving a diverse panel of international experts, we sought to develop a series of consensus statements that represent expert opinion and could serve as a guiding framework for surgical education and policy worldwide.

## Materials and methods

Study design and timeline

A modified Delphi consensus process was conducted over 18 months, from January 2023 to June 2024, to achieve a global consensus on the role of advanced technologies in surgical training.

Modifications to the Delphi Process

The modifications involved combining a structured quantitative scoring process with three virtual consensus meetings to allow for real-time debate, immediate refinement of statements based on qualitative feedback, and acceleration of the traditional, anonymous multi-round approach. This approach maximized expert engagement and facilitated the convergence of opinion.

Expert panel recruitment and logistics

A 23-member expert panel was formed, comprising surgeons, surgical educators, and health policy experts from 16 different countries across six WHO regions. 

Selection Process and Potential Bias

Panel members were identified via the Artificial Intelligence Surgery (AIS) Editorial Board and a targeted literature review, then specifically invited based on their high-level expertise and publications in surgical training, advanced technologies, and AI. This method constitutes purposive sampling, which, while ensuring deep subject matter expertise, is acknowledged as introducing a potential selection bias toward individuals with an existing interest in and commitment to advanced surgical technology.

Equitable Participation

To ensure equitable participation across global time zones and varying internet infrastructure:

Time zone accommodation: All virtual consensus meetings were held at two distinct, widely separated times (e.g., morning and afternoon EST) to accommodate participants from the Americas, Europe, and Asia.

Asynchronous feedback: Detailed meeting minutes and recordings were distributed immediately after both sessions. Experts who could not attend the live meetings or faced connectivity issues were asked to review the documents and submit their comments asynchronously via email within a specified window.

Consensus process phases

The consensus-building process was structured in three distinct phases, moving from initial content generation and validation to iterative refinement and final consensus confirmation:

Phase 1: Questionnaire Development

A steering committee of five experts developed a 25-item questionnaire based on a comprehensive literature review. This committee, comprising members of the AIS Editorial Board and Task Force, developed and internally validated the questionnaire through consensus among the subject matter experts before its dissemination to the larger panel.

External Validation and Pilot Testing

The draft 25 statements were subjected to pilot testing by a small group of three independent, non-panel surgical experts for clarity, ambiguity, and relevance testing. Statements were refined based on this feedback to ensure they were measurable and suitable for the 5-point Likert scale, enhancing content validity and reliability. The questionnaire was designed to elicit opinions on five key topics: the global burden of surgical disease, simulation training, telementoring, AI in training, and policy implications. All questions were presented on a 5-point Likert scale, and participants were also given the opportunity to provide qualitative comments.

Phase 2: Consensus Meetings

Three virtual consensus meetings were held to ensure equitable participation and constructive dialogue. The purpose of these meetings was to discuss the quantitative results from the first round of the questionnaire, debate statements that did not initially reach consensus, and refine the wording of certain statements based on qualitative feedback. The revised statements and aggregated anonymous results were then sent back to the panel.

Phase 3: Final Analysis

After the third meeting, a final round of the questionnaire was administered to confirm the final consensus on all statements.

Consensus Definition and Statistical Analysis

Consensus was pre-defined as a threshold of or greater agreement, combining "agree" and "strongly agree" responses. This threshold was chosen a priori to ensure a strong supermajority of agreement, consistent with rigorous standards in high-stakes clinical and medical education consensus studies, thereby enhancing the authority and robustness of the final report.

The responses were analyzed using descriptive statistics, including median score and interquartile range (IQR) to assess the strength and variance of agreement, and qualitative data from open-ended responses and the virtual meetings were analyzed using a thematic approach and used solely to refine the wording of the consensus statements and inform the final Discussion section. A single researcher on the steering committee compiled the recurring themes for presentation to the larger panel for debate. A subgroup analysis comparing the final scores of high-income country (HIC) participants versus LMIC participants was performed for key statements related to feasibility and cost.

## Results

Panel demographics and consensus summary

The demographic characteristics of the 23-member expert panel who participated in the Delphi consensus process are summarized in Table 1. Consensus (≥80% agreement) was reached on 13 of the 25 statements. The full results table (Table 2) includes the median score and IQR for all 25 statements to provide a clearer picture of the strength and certainty of agreement. Conversely, consensus was not reached on the remaining 12 statements, indicating areas of non-uniform expert opinion. The results are presented below, organized by the five key themes.

**Table 1:** Demographics of the Artificial Intelligence Surgery Editorial Board and Survey Participants WHO: World Health Organization

Characteristic	Number of Participants (n)	Percentage (%)
Total Participants	23	100%
Number of Countries Represented	16	
WHO Region Representation		
African Region	2	8.70%
Americas Region	6	26.10%
Eastern Mediterranean Region	3	13.00%
European Region	8	34.80%
South-East Asia Region	2	8.70%
Western Pacific Region	2	8.70%
Professional Role		
Surgeon/Clinician	15	65.20%
Surgical Educator/Researcher	5	21.70%
Health Policy Expert	3	13.00%
Years of Experience in Surgical Education		
5-10	7	30.40%
11-20	10	43.50%
>20	6	26.10%

Key consensus findings by theme

Global Burden of Surgical Disease

The panel unanimously agreed (100% agreement) that there is a significant, unaddressed global surgical burden, with a particularly acute lack of surgical capacity in LMICs [1,2]. There was also a strong consensus (95% agreement) that advanced technologies could play a pivotal role in augmenting existing training programs and scaling up the surgical workforce. The experts agreed that these technologies should be viewed as a tool to bridge the skill gap, not as a replacement for traditional, hands-on training.

Simulation Training

A strong consensus (91% agreement) was reached on the need to integrate simulation technologies into modern surgical curricula globally. This consensus included a wide range of technologies, from low-cost bench-top models to sophisticated VR simulators [3,4,7]. The panel agreed that simulation should be standardized to ensure that trainees meet competency benchmarks before transitioning to patient care. Furthermore, there was consensus on the importance of making accessible and affordable simulation tools a priority to promote equitable training across all regions.

Telementoring

The panel overwhelmingly agreed (95% agreement) that telementoring - the use of real-time audio and video to guide a surgeon remotely - is a valuable tool [8,9]. The consensus highlighted its potential to provide expert guidance during complex procedures, offer immediate feedback, and reduce the need for travel, thereby making expert mentorship more accessible in LMICs [10,11]. There was also consensus that telementoring could be a cost-effective way to enhance surgical capacity in remote and under-resourced areas.

Artificial Intelligence (AI) in Surgical Training

A robust consensus (95% agreement) was reached on the potential of AI to enhance surgical training [12,13,14]. The experts agreed that AI-powered systems could provide objective and personalized feedback on surgical performance, moving beyond subjective assessments [15,16]. Specific areas where AI was seen as highly promising included automated skill assessment, identification of procedural errors, and the provision of targeted, real-time coaching. The consensus also noted the need for validating AI-based tools to ensure their accuracy and reliability before widespread adoption [17].

Policy and Implementation

The panel agreed that for these technologies to be successfully implemented, robust policy frameworks are essential. A consensus was reached (87% agreement) on the need for institutional support, dedicated funding, and clear ethical guidelines to govern the use of AI and other technologies in training [18,19,20,21,22]. The experts emphasized that these policies should address data privacy, patient consent, and the equitable distribution of resources to avoid exacerbating existing global health disparities.

Areas of Non-Consensus

Consensus (>80% agreement) was not reached on 12 of the 25 statements, indicating specific areas of uncertainty or disagreement among the panel. The statements that failed to achieve the threshold were primarily focused on:

Mandated curricular changes: Specific requirements for technology integration into national curricula often showed lower agreement due to regional variability in regulatory authority.

Specific funding mechanisms: Statements regarding mandated government funding or the cost-sharing ratio between public and private entities did not achieve consensus, highlighting challenges in financial implementation.

Regulatory oversight: Statements related to the specific timing and responsibility for the continuous re-validation of AI models (e.g., every six months) revealed high variability in expert opinion, likely due to a lack of current definitive guidance.

Table 2 presents the key consensus statements agreed upon by the expert panel, categorized by the five themes of the Delphi process. Consensus was defined as an >80% agreement rate.

**Table 2:** Global Consensus Statements on Advanced Technologies in Surgical Training

Theme	Statement	N (%)
Global Burden of Surgical Disease	"There is a significant, unaddressed global surgical burden, particularly in low- and middle-income countries (LMICs)."	23 (100%)
	“Advanced technologies should be viewed as a vital tool to augment existing training programs and scale up the surgical workforce.”	22 (95%)
Simulation Training	"Simulation technologies, ranging from low-cost task trainers to advanced virtual reality (VR), are essential components of modern surgical curricula."	21 (91%)
	“Simulation-based training should be standardized to ensure trainees meet specific competency benchmarks before operating on patients.”	21 (91%)
	“It is a priority to develop accessible and affordable simulation tools to promote equitable training across all regions.”	21 (91%)
Telementoring	“Telementoring is a valuable tool for providing expert guidance during complex procedures and is particularly useful in remote areas.”	22 (95%)
	"Telementoring can effectively reduce geographical barriers and make expert mentorship more accessible, especially in LMICs."	22 (95%)
Artificial Intelligence (AI) in Training	"AI has the potential to provide objective, personalized feedback on surgical performance, moving beyond subjective assessments."	22 (95%)
	“AI-powered systems can assist in automated skill assessment and the identification of procedural errors in real-time.”	22 (95%)
	“AI-based tools for surgical training must be rigorously validated to ensure accuracy and reliability before widespread adoption.”	22 (95%)
Policy and Implementation	"The successful implementation of these technologies requires strong institutional support, dedicated funding, and clear policy frameworks."	20 (87%)
	"Policies and guidelines must address ethical considerations, including data privacy and patient consent."	20 (87%)
	“It is critical to ensure that the adoption of advanced technologies does not widen existing global health disparities.”	20 (87%)

## Discussion

Overview and foundational framework

The consensus reached by this diverse panel of international experts provides a foundational framework and prioritized agenda for the future of surgical training, grounded in expert opinion. The findings affirm that advanced technologies are not just a luxury but are expert-affirmed as a necessity for addressing the global surgical burden, particularly in LMICs [1,2]. The unanimous agreement on the existence of a significant surgical disparity sets the stage for a collective effort to leverage technology for equitable training.

Key areas of consensus

The consensus on simulation and telementoring reinforces their established value but also emphasizes the need for broader adoption, especially in under-resourced settings. These consensus points are supported by empirical validation studies showing the construct and predictive validity of key simulation platforms and the technical feasibility of telementoring in remote pilot programs.

The panel's strong support for AI in surgical training is a significant finding. It signals a readiness among surgical educators to embrace a new era of training that prioritizes the potential of objective, data-driven assessment and personalized learning. This potential is supported by empirical evidence from studies that have validated AI and machine learning algorithms in providing objective, automated assessments and reducing inter-rater variability in skill scoring, which is the mechanism that enables feedback democratization. The expert-affirmed potential for AI to democratize access to high-quality feedback is particularly promising for training programs that lack sufficient expert mentors.

The integration of AI in surgical training presents critical ethical challenges alongside its opportunities. The most critical risks identified were data privacy and algorithmic bias. Ensuring trustworthy AI algorithms necessitates development regulated by medical ethics and core human values, prioritizing data privacy and transparency (including compliance with regulations like GDPR and HIPAA [23]) while minimizing bias [18]. A concrete mitigation strategy for algorithmic bias is the mandatory use of diverse, multicentric datasets that include high representation from LMICs and diverse demographics, subject to regular, independent audits [22,24,25].

The discussion also highlighted the integration of these technologies into the curriculum, including the rise of robotic surgery [26]. A crucial aspect of this is the focus on equity, which includes considering the specific needs of medical students and surgical residents with physical disabilities to ensure equitable access to training [27]. The personal account of the surgeon completing training after a neck injury serves as a powerful illustration that advanced technologies can overcome physical limitations, validating the consensus statement on a more inclusive surgical workforce. Furthermore, advanced technologies can also play a role in improving surgeons' well-being by mitigating occupational injuries, which is an often-overlooked aspect of surgical practice [28]. This is all part of a broader effort to develop and validate virtual teaching methods for minimally invasive surgical skills [29].

Policy, implementation, and practical challenges

The consensus on policy and implementation highlights the practical challenges ahead. Simply developing the technology is not enough; its successful integration requires a supportive ecosystem of institutional leadership, dedicated funding, and ethical oversight. Furthermore, the widespread clinical adoption is contingent upon achieving full regulatory clearance (e.g., FDA, CE marking) from appropriate bodies, and we call for accelerated and harmonized validation pathways for surgical training technologies.

Achieving this supportive ecosystem in LMICs, where institutional leadership and funding are constrained, requires prioritizing low-cost policy changes (e.g., protecting virtual training time) and strategic investment in essential infrastructure (e.g., dedicated low-bandwidth internet) over large-scale, high-cost technology purchases. The experts’ call for policies that prevent the widening of health disparities is a crucial reminder that technology must be deployed thoughtfully and with a focus on equity. The HIC vs. LMIC subgroup analysis confirmed that while the LMIC participants are highly supportive of the technology's potential, they exhibited significantly lower median scores on statements related to immediate feasibility and funding capacity, underscoring the necessity of contextual limitations.

These consensus points directly link back to the practical limiting factors of resource, cost, and compliance protocols, underscoring that the recommendations’ successful adoption is contingent upon overcoming these barriers. Conversely, the 12 statements that failed to reach consensus are primarily centered on implementation specifics (e.g., mandatory funding mechanisms, granular regulatory timelines), which confirms that these areas are the current, unresolved boundaries of expert knowledge and should be the focus of future research.

This consensus project provides a solid foundation for future research and implementation efforts. It suggests that the path forward for surgical training involves a hybrid model that combines traditional methods with innovative technologies, all guided by a shared foundational agenda to improve surgical care for everyone.

Limitations

This study has several limitations. The expert panel, while diverse, consisted of only 23 members from 16 countries, which may not comprehensively capture global perspectives. Specifically, the limited proportional representation from LMICs means the findings reflect a high-level, international policy perspective rather than a primary consensus rooted in the day-to-day realities of those specific regions. Furthermore, the selection process, based on existing expertise in technology, introduced a potential selection bias. The Delphi process, by design, focuses on establishing consensus rather than implementing or validating the technologies in a clinical setting. Therefore, the study's findings are based on Level V (expert opinion) evidence and inherently lack empirical data on the effectiveness of the proposed solutions. Furthermore, while the study identified critical ethical challenges and policy needs, it did not provide a definitive framework for resolving issues such as data privacy, algorithmic bias, or equitable resource distribution. The successful implementation of these technologies in practice remains contingent on future efforts to secure funding, develop robust policies, and validate the efficacy of AI-based tools.

## Conclusions

This global consensus, representing experts from a wide range of countries and specialties, provides a set of foundational consensus principles and a guiding framework derived from international expert opinion for the future of surgical training. The findings highlight the expert-affirmed critical role of advanced technologies - including AI, simulation, and telementoring - in addressing the global surgical burden and enhancing the quality of surgical education. The panel believes these tools could help reduce the global surgical burden and promote a more inclusive surgical workforce; however, this potential impact needs to be confirmed through empirical research. The consensus not only affirms the potential of these tools but also underscores the necessity of a standardized approach to their integration into global training curricula.

Successful implementation of these technologies requires a concerted, collaborative effort among primary stakeholders: policy makers, industry developers, and surgical educators. It necessitates targeted investment from institutions and governments, robust policy frameworks, and clear ethical guidelines. This includes advocating for concrete mechanisms such as International Working Groups (for harmonizing standards) and Public-Private Partnerships (for subsidized LMIC deployment). These measures are crucial to promote equitable resource distribution and to ensure that the adoption of advanced technologies does not worsen existing global health disparities, while also paving the way for a more inclusive surgical workforce. By harnessing these innovations to improve surgical education and, ultimately, patient care for all, the global surgical community can advance toward a data-driven future. Nevertheless, since these findings reflect expert consensus rather than empirical evidence, future research is essential to validate the effectiveness of the proposed solutions.
